# Complete relief by omalizumab in eruptive pruritic papular porokeratosis

**DOI:** 10.1016/j.jdcr.2024.04.030

**Published:** 2024-04-28

**Authors:** Hongxia Jia, Ruzeng Xue, Liwei Ran

**Affiliations:** a Department of Dermatology, Beijing Chao-Yang Hospital, Capital Medical University, Beijing, China; b Department of Dermatology, Dermatology Hospital, Southern Medical University, Guangzhou, China

**Keywords:** eruptive pruritic papular porokeratosis, omalizumab

## Introduction

Eruptive pruritic papular porokeratosis (EPPP) was first proposed by Kanzaki et al in 1992^1^ and named “Inflammatory disseminated superficial porokeratosis (Inflammatory DSP)” by Tanaka et al in 1995.^2^ EPPP is a subtype of porokeratosis (PK). It is clinically manifested in 3 distinct phases: an asymptomatic PK rash that can last for many years, a sudden exacerbation with severe pruritus, and a papular eruption that resolves after a few months. This intense itching seriously affects patients' quality of life. Herein, we report the first case of successful relief of EPPP treated with omalizumab.

## Case report

**Fig 1:**
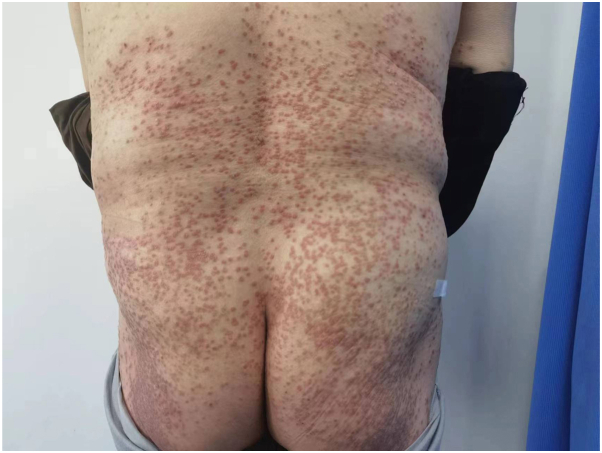
Dense keratinized papules on the trunk, 1 to 4 mm in diameter and varied from *red* to *dark red* or *brown* in color. Some lesions appeared as central atrophy with raised *borders*, some were firm papules, and some were covered by thin scales.

**Fig 2:**
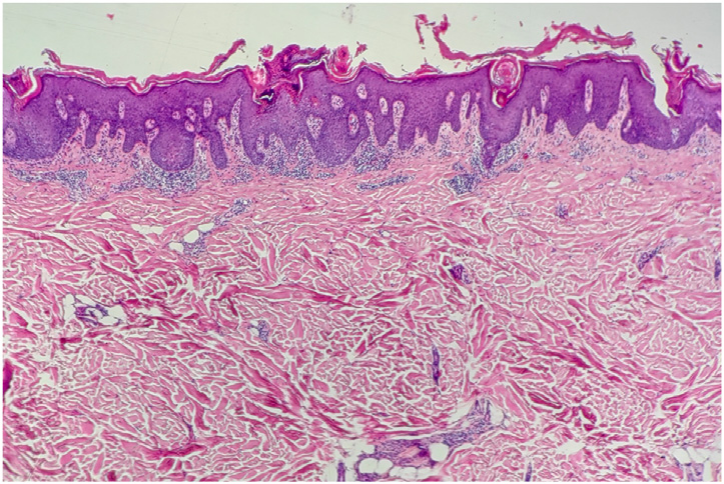
Histopathology demonstrated cornoid lamella formation, hypogranulosis, vacuolation, and dyskeratosis in keratinocytes, perivascular and interstitial lymphocytes, eosinophils and mast cells infiltration were observed (×100).

**Fig 3:**
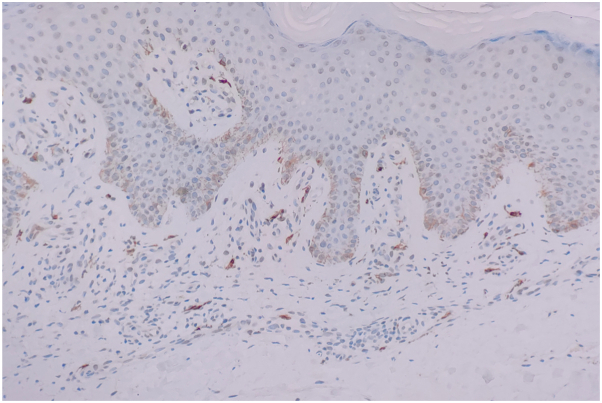
Immunohistochemistry revealed positive expression of CD117.

**Fig 4:**
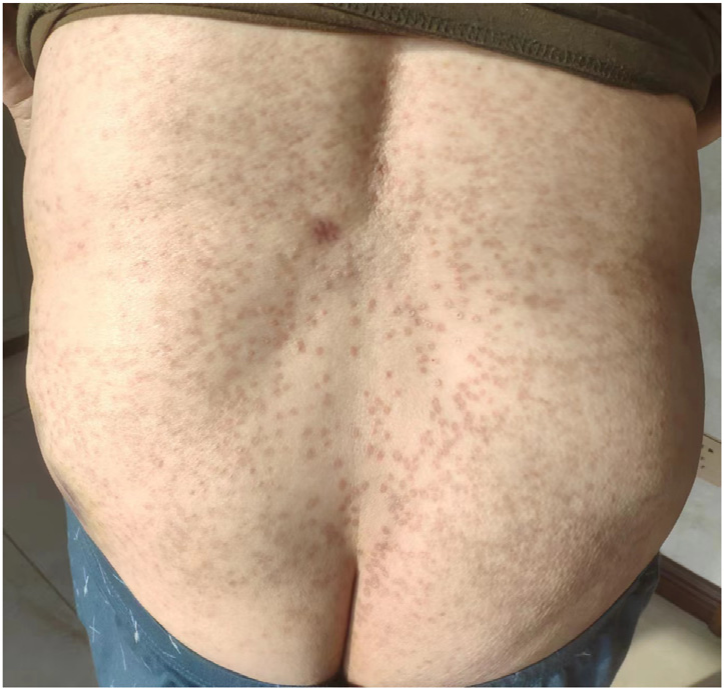
The patient's skin lesions subsided significantly after treatment with omalizumab.

A 69-year-old male diagnosed with EPPP had a 40-year history of PK only experiencing itching after sun exposure. In the past 10 months, the rash deepened and increased in color, accompanied by severe itching following the COVID-19 infection. There were many densely keratinized papules on the trunk and extremities, ranging from 1 to 4 mm in diameter and varying from red to dark red in color. Some lesions appeared with central atrophy and raised borders, some were firm papules, and some were covered by thin scales (Fig 1). Both his father and brother had a history of PK. The patient had a history of chronic spontaneous urticaria for 5 years and no history of cancer. Histopathology demonstrated cornoid lamella formation, hypogranulosis, vacuolation, and dyskeratosis in keratinocytes. Additionally, perivascular and interstitial lymphocytes, eosinophils, and mast cells infiltration were observed (Fig 2). Immunohistochemistry revealed positive expression of CD117 (Fig 3). Laboratory tests showed no abnormalities. Based on the patient’s history, clinical manifestations, and histopathological examination, EPPP diagnosis was confirmed. In the past, the patient was treated with multiple medications, including loratadine, cetirizine, and injections of betamethasone. Unfortunately, these treatments failed. Due to the obvious pruritus, eosinophils and mast cells infiltration in histopathology, as well as the patient’s history of chronic spontaneous urticaria, a 300-mg omalizumab injection was administered. The patient's skin lesions significantly subsided (Fig 4), and the itching nearly disappeared following the initial injection. Consequently, the patient was administered 2 additional omalizumab injections of 300 mg, each spaced 4 weeks apart. To date, no recurrence of symptoms has been observed.

## Discussion

Current treatments for EPPP include glucocorticoids, antihistamines, vitamin A derivatives, and others, all of which have shown some efficacy. However, patients with severe itching do not always achieve ideal results.^3^ The mechanism of itching in patients with EPPP is not well understood, but lymphocytes and eosinophil infiltration can be observed in EPPP. This lymphocyte inflammation and eosinophilic activity may contribute to the progression of lesions.

Omalizumab has been approved for severe allergic asthma and chronic spontaneous urticaria. More recently, omalizumab has been used off-label for some diseases in which IgE may or certainly plays an important role, such as allergic rhinitis, allergic reaction, bullous pemphigoid, and others.^4^

In our case, there were some perivascular and interstitial mast cell infiltration in the superficial dermis, so we believe that the possible principle of omalizumab's effectiveness in EPPP treatment is its ability to stabilize mast cell membranes, but more studies need to be conducted.

## Conflicts of interest

None disclosed.
